# Predictive Value of IL-6 and Lymphocyte Subsets for Death in Children With Influenza-Associated Encephalopathy A Retrospective Study

**DOI:** 10.1155/mi/5564727

**Published:** 2024-11-23

**Authors:** Ruiyang Sun, Xue Zhang, Jiapu Hou, Wanyu Jia, Peng Li, Daobin Wang, Shuqin Fu, Chunlan Song

**Affiliations:** ^1^Emergency Department, Henan Province Engineering Research Center of Diagnosis and Treatment of Pediatric Infection and Critical Care, Children's Hospital Affiliated to Zhengzhou University, Zhengzhou, China; ^2^Pediatric Department, Zhecheng County People's Hospital, Shangqiu, China

**Keywords:** death, IL-6, influenza-associated encephalopathy, lymphocyte subsets, receiver operating characteristic

## Abstract

**Objective:** This study aims to investigate the predictive value of interleukin-6 (IL-6) and lymphocyte subsets for death in children with influenza-associated encephalopathy (IAE).

**Patients:** This study included 76 children with IAE who were divided into a death group and a survival group. The differences in the levels of IL-6 and lymphocyte subsets between the two groups were analyzed, and the predictive value of these two parameters was compared by receiver operating characteristic (ROC) curve analysis.

**Results:** The level of IL-6 and the percentage of natural killer (NK) cells in the death group were higher than those in the survival group (*p*  < 0.05). The percentage of CD4^+^ T cells and CD4^+^/CD8^+^ levels in the death group were lower than those in the survival group. ROC curves were used for analysis, and the area under the curves (AUCs) of IL-6, the percentage of CD4^+^ T cells, the percentage of CD4^+^/CD8^+^, and the percentage of NK cells were 0.812, 0.810, 0.740, and 0.706, respectively. The AUC of the combination of these four metrics was 0.870. There was a little difference in the efficacy of the four clinical indicators, and the predictive efficacy of the combined test was higher than that of the single test.

**Conclusion:** The IL-6 concentration, percentage of CD4^+^ T cells, percentage of NK cells, and CD4^+^/CD8^+^ have predictive value for death in children with IAE, and the combination of these four metrics has improved the predictive value.

## 1. Introduction

Influenza-associated encephalopathy (IAE) is a syndrome that presents with central nervous system dysfunction during the acute phase of influenza [1]. The clinical manifestations of IAE are neurologic symptoms such as convulsions, coma, and motor or sensory deficits, which usually appear 24 h or 2–3 days after the onset of influenza symptoms [2]. The incidence of IAE is low, ranging from 0.6 to 1.1 cases/100,000 in people under 15 years of age and 1.2–2.9 cases/100,000 in people under 4 years of age; however, the mortality rate of IAE is high, ranging from 20% to 30% [3]. Therefore, early identification of high-risk factors for death in children with IAE and active diagnosis and treatment are highly important for reducing mortality and improving the prognosis of IAE.

Interleukin-6 (IL-6) is a four-helix protein consisting of 184 amino acids that is produced as a cytokine by monocytes and macrophages following pathogen invasion [4]. It can transmit signals of invasion or damage to the body and quickly activate the body's defense system [5]. Depending on the type and severity of the disease, the level of IL-6 varies greatly; the serum IL-6 concentration in healthy people is generally less than 4 pg/mL, but in chronic diseases, the level can increase dozens or even hundreds of times. The concentration can exceed 1000 pg/mL during septic shock or “cytokine storm” [6]. Changes in cytokine levels in patients correlate with disease severity and prognosis and are preceded by changes in clinical symptoms [7].

The lymphocyte subsets included natural killer (NK) cells, B cells, CD4^+^ T cells (CD3^+^ and CD4^+^), and CD8^+^ T cells (CD3^+^ and CD8^+^). Changes in lymphocyte subsets reflect the immune status of the body, and a disturbed immune response may lead to local or systemic damage [8]. One study revealed a decrease in CD4^+^ T lymphocytes and a decrease in CD4^+^/CD8^+^ in patients with severe influenza pneumonia [9]. Both CD4^+^ and CD8^+^ T cells contribute to virus clearance during primary or secondary influenza virus infection [10]. By producing large numbers of cytokines and coordinating with other immune and nonimmune cells, CD4^+^ T cells influence the degree of lung inflammation and damage caused by influenza virus infection [11]. CD4^+^ T cells stimulate B-cell activation and differentiation and help CD8^+^ T cells differentiate into cytotoxic effects and memory cells. In addition, CD4^+^ T cells with cytotoxic potential have the same ability to kill infected cells. CD8^+^ T cells can recognize invading cells, induce cell apoptosis, and produce proinflammatory cytokines to inhibit viral replication [12]. Influenza virus can induce lymphocyte apoptosis and inhibit lymphocyte stem cell proliferation in the bone marrow, resulting in a decrease in lymphocytes [13]. NK cells can directly kill and lyse virus-infected cells, and their function is regulated by signals transduced by self-expressed activating/inhibitory receptors. When the virus invades the cell and attenuates the signal that inhibits NK cell activation, NK cells activate and kill the infected cells [14]. In addition, infected host cells release type I interferon, which enhances the cytotoxic effects of NK cells [15]. NK cells also produce immune interferon, tumor necrosis factor (TNF), IL-10, and chemokines, which regulate immune and inflammatory responses.

However, studies on changes in IL-6 and lymphocyte subsets in children who die from IAE are rare. Alterations in IL-6 and lymphocyte subsets reflect the inflammatory profile and immune status of the organism, and we hypothesized that these indicators may be associated with death in children with IAE. Therefore, this study explored the changes in IL-6 levels and lymphocyte subsets in children who died from IAE and analyzed the clinical benefits of these indicators in predicting death in children with IAE.

## 2. Methods

### 2.1. Objects of the Study

The participants in this study were children diagnosed with IAE at the Affiliated Children's Hospital of Zhengzhou University between January 2018 and September 2023; these patients were divided into a death group and a survival group based on the final clinical outcome of the children. This study was approved by the Medical Ethics Committee of Children's Hospital Affiliated to Zhengzhou University with the ethical approval number 2023-K-129, and no private information of the children will be exposed in this paper.

### 2.2. Inclusion and Exclusion Criteria

The diagnostic criteria for IAE were as follows: (1) The children were diagnosed with influenza according to the Expert Consensus on the Diagnosis and Treatment of Influenza in Children (2020 Edition) [16]; (2) children with neurological symptoms such as motor or sensory dysfunction, convulsions, and coma within 24 h or 2–3 days of influenza virus infection; (3) children with coinfections of varicella virus, rotavirus, parainfluenza virus, human herpesvirus-6, measles virus, rubella, coxsackie virus, herpes simplex virus, or other pathogens that may cause encephalopathy were excluded; and (4) exclude children with meningitis, myelitis, febrile convulsions, or other diseases causing encephalopathy or encephalitis.

The inclusion criteria were as follows: (1) age <18 years and (2) confirmed diagnosis of IAE. The exclusion criteria were as follows: (a) children whose clinical data were missing; (b) children who were transferred to other hospitals during the course of treatment; (3) children with other types of previous encephalopathies, such as neonatal hypoxic–ischemic encephalopathy, hepatic encephalopathy, or bilirubin encephalopathy; and (4) children with autoimmune dysfunction diseases, acquired immunodeficiency diseases, or glucocorticoid hormone and immunosuppressant use before influenza infection were excluded.

### 2.3. Data Collection

We utilized an electronic medical record system to collect age, sex, prognosis, and laboratory findings of children with IAE, as follows: IL-6 concentration, percentage of CD4^+^ T cells, percentage of CD8^+^ T cells, percentage of NK cells, and CD4^+^/CD8^+^.

### 2.4. Determination of Sample Size and Test Level

The sample size included in our study was small, and the probabilities of type I and type II errors were high. Since the purpose of our statistical analysis was to determine the indicators of differences between the group of children with IAE who died and the group who survived, we used a test level of *α* = 0.05 to reduce the probability of type I errors during the statistical analysis. We used Power Analysis and Sample Size (PASS) 2023 (version 23.0.2.) to estimate the sample size. With *α* = 0.05, area under the curve (AUC) = 0.8, and a 2:1 ratio between the survival group and death group, the sample size of the survival group was 48, and that of the death group was 24. Our sample size is similar to the calculated theoretical value, so it is reasonable to conduct a statistical analysis at the test level *α* = 0.05.

### 2.5. Data Analysis

We used Statistical Product and Service Solutions (SPSS) 26.0 to analyze the data of the two groups of children with IAE. The normally distributed quantitative data are expressed as the mean ± standard deviation (*χ* ± s), and the *t* test was used for comparisons between groups. Quantitative nonnormally distributed data are expressed as medians (M) and interquartile intervals (Q1 and Q3), and the Mann‒Whitney *U* test was used for comparisons between groups. The chi-square test was used for qualitative data. We used MedCalc software to plot the receiver operating characteristic (ROC) curves to evaluate the accuracy of each index and to determine the optimal cutoff value. The AUC of the ROC curve for each indicator was compared using a nonparametric method. *p* < 0.05 was considered to indicate statistical significance.

## 3. Results

### 3.1. Demographic and Clinical Characteristics of Children With IAE

A total of 76 children with IAE who were hospitalized in the Affiliated Children's Hospital of Zhengzhou University from January 2018 to September 2023 were included in this study after screening; the male‒female ratio was 1.71:1, and the median age was 4 years. There were 57 cases of influenza A infection, 18 cases of influenza B infection, and 1 case of combined A and B infection; there were 43 cases of cough, 15 cases of dyspnea, 31 cases of vomiting, 5 cases of diarrhea, and 18 cases of underlying diseases; and there were 57 cases of respiratory complications, 20 cases of circulatory complications, 27 cases of digestive complications, and 15 cases of hematologic complications.

### 3.2. Comparison of Baseline Characteristics and Indicators Between the Two Groups

There were 59 cases in the survival group and 17 cases in the death group. At present, there is no specific treatment for IAE. There were no statistically significant differences between the two groups in terms of age, sex composition, underlying disease, number of days with fever, type of influenza, fever, cough, dyspnea, vomiting, diarrhea, or percentage of CD8^+^ T cells. IL-6 levels and the percentage of NK cells were lower in the survival group than in the death group (*p* < 0.05), and the percentage of CD4^+^ T cells and the ratio of CD4^+^/CD8^+^ in the survival group were higher than those in the death group (*p* < 0.05). The specific data are shown in Table 1.

### 3.3. ROC Curves for Each Indicator and Analysis Results

**Figure 1:**
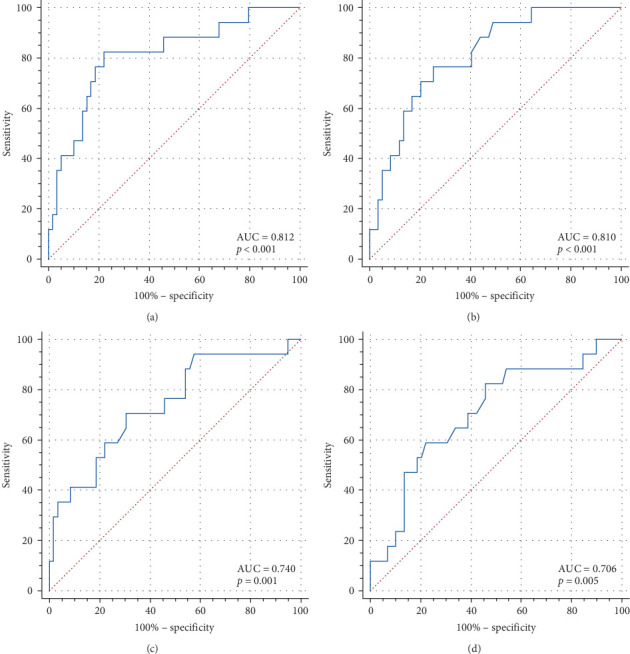
ROC curves of IL-6, CD4^+^ T cell, NK cell, and CD4^+^/CD8^+^ predicting death in children with IAE: (a) ROC curve for IL-6, (b) ROC curve for the percentage of CD4^+^ T cells, (c) ROC curve for the percentage of NK cells, and (d) ROC curve for CD4^+^/CD8^+^. IAE, influenza-associated encephalopathy; IL-6, interleukin-6; NK, natural killer; ROC, receiver operating characteristic.

**Figure 2:**
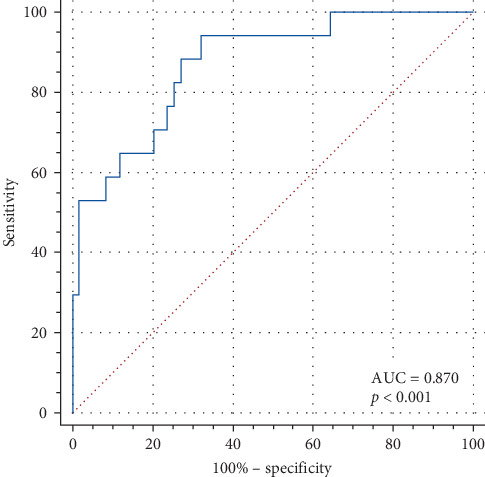
ROC curves for the combined assay. ROC, receiver operating characteristic.

Differences in laboratory test results between the two groups were used as indicators of death in children with IAE. ROC curve was drawn to analyze its predictive value (Figure 1). The combined IL-6 concentration, percentage of CD4^+^T cells, percentage of NK cells, and CD4^+^/CD8^+^ were used to predict death in children with IAE, and ROC curves were plotted to validate their effects (Figure 2). The AUC values, 95% confidence intervals (CIs), sensitivities, and specificities of the ROC curves for IL-6, CD4+ T cells, NK cells, CD4^+^/CD8^+^, and the composite indices are shown in Table 2. Compared with the individual ROC curves of the above four indicators, the AUC of the combined assay was higher than the percentage of CD4^+^ T cells (*p*=0.039), the percentage of NK cells (*p*=0.035), and the AUC of CD4^+^/CD8^+^ (*p*=0.041). Similarly, the AUC of the combined assay was higher than the AUC of IL-6, but the difference was not statistically significant (*p* > 0.05).

## 4. Discussion

IAE is a leading cause of death in children with influenza [17]. The pathogenesis of IAE has not yet been determined, and the “cytokine storm hypothesis,” which states that a large number of IL-6 and other cytokines in children with influenza promote the occurrence of IAE, is widely accepted internationally [1]. It has been suggested that the degree of brain imaging changes in children with IAE correlates with prognosis [18], but after the appearance of imaging changes in children, their disease is often more serious. In comparison, alterations in cytokines and lymphocyte subsets precede clinical symptoms and pathological changes in children. Therefore, we used IL-6 and lymphocyte subsets as indicators of mortality in children with IAE and studied their relationships with mortality in these children.

IL-6 has a variety of biological effects that regulate the immune system and affect its function. Previous studies have shown that IL-6 levels are greater in children with IAE than in children with influenza without neurologic complications and that severity is positively correlated with inflammatory factor levels [19]. Aiba et al. [20] reported that, during the disease course, children with IAE whose blood IL-6 levels exceeded 15,000 pg/mL had a clinical outcome of death, and the lower the blood IL-6 level was, the less severe the central nervous system sequelae were; therefore, they concluded that high levels of IL-6 in the pathological state not only led to an increased inflammatory response in the body but also had a neurotoxic effect. These factors may account for the development of IAE in children with influenza as well as the worsening of neurologic symptoms. IL-6 can be used to predict the severity of illness or death in influenza patients [21]. Elevated IL-6 is also a predictor of intensive care unit admission in influenza patients [22]. It has been found that IL-6 prevents apoptosis in virus-infected cells by increasing the expression of prosurvival molecules, such as Bcl-2 and Bcl-xL, through the signal transducer and activator of transcription 3 (STAT3) and nuclear factor kappa B (NF-*κ*B) signaling pathways [23]. In addition, IL-6 can induce the expression of vascular endothelial growth factor in epithelial cells, increase vascular permeability, and decrease myocardial contractility, thus promoting multiple organ damage [24]. In our study, we found that IL-6 levels were significantly higher in the death group. Thus, we used the IL-6 level as an indicator to predict death in children with IAE.

CD4^+^ T cells inhibit viral replication by directly killing or secreting interferon and TNF, whereas CD8^+^ T cells directly kill virus-infected cells by specifically recognizing antigens [25]. Previous studies have shown that patients with severe influenza have reduced CD4^+^ T cells and reduced CD4^+^/CD8^+^ [26]. In our study, the percentage of CD4^+^ T cells was significantly lower, and the percentage of CD4^+^/CD8^+^ was lower in the death group than in the control group, suggesting that the number of lymphocytes in the bodies of children with IAE was reduced by the influenza virus, especially CD4^+^ T cells. A reduction in CD4^+^ T cells can reduce the function of CD8^+^ T cells, leading to a decrease in the body's immune function and eventually death. We found that the CD4^+^ T cell percentage and CD4^+^/CD8^+^ can be used as prognostic predictors of death in children with IAE and that children with IAE have an increased risk of death when their CD4^+^ T cell percentage is less than 31.1% or when their CD4^+^/CD8^+^ is less than 0.33.

It has been shown that NK cells play dual roles during influenza virus infection; when viral loads are low, NK cells play an important role in limiting early viral spread, whereas in severe influenza with excessive viral loads, NK cells exacerbate tissue damage and pathological responses [27]. Lang et al. [28] research had found that activated NK cells in mice infected with lymphocytic choriomeningitis virus can specifically clear CD4^+^ T cells, thereby inhibiting the immune response of CD8^+^ T cells to the virus. By studying an influenza mouse model, it was concluded that NK cells could induce harmful inflammation in the lung and lead to the death of influenza mice in addition to the above mechanisms [29]. The level of immature NK cells in patients with severe COVID-19 is significantly elevated, and immature NK cells can secrete a large number of cytokines to aggravate the “cytokine storm” in the body. Moreover, the ability of immature NK cells to kill virus-infected cells is reduced so that the virus can survive for a long period of time in the body [30]. In our study, we found that the percentage of NK cells in the death group was greater than that in the survival group and that children with IAE were more likely to have a fatal outcome when the percentage of NK cells was greater than 12.88%. These findings suggest that NK cells in children with severe IAE can further reduce the percentage of CD4^+^ T cells and weaken the body's ability to clear viruses and that NK cells can promote the production of TNF-α and other cytokines, aggravate the “cytokine storm” in children, and ultimately promote their death.

We compared the diagnostic efficacy of four indicators, IL-6, the percentage of CD4^+^ T cells, CD4^+^/CD8^+^, and percentage of NK cells, for predicting death in children with IAE and found that there was little difference in the predictive value of each of the four indicators. We found that the predictive value of the four metrics combined was superior to that of CD4^+^ T cells, CD4^+^/CD8^+^, and NK cells alone but not to that of IL-6 alone. IAE has a high mortality rate, so the high sensitivity of the combined assay facilitates the early detection of children with IAE who are prone to death and helps to reduce the mortality rate of children with IAE. In addition, the combined assay can more comprehensively reflect the immune status of children with IAE, which can provide a reference for evaluating the disease progression and severity of children with IAE, and contribute to the stratified risk management of children with IAE.

## 5. Limitations

There are several limitations to our study. This is a single-center retrospective study with a small sample size and is prone to type II error. We did not further study whether there were differences in IL-6 and lymphocyte subsets in different subtypes of IAE. The immune status of IAE children with different influenza types was not analyzed. The data in our study came from the diagnosis and treatment process of hospitalized patients, and the collection time of patient samples and the underlying disease status may affect the determination of the results. Our findings need to be validated in multicenter, large sample sizes, and prospective studies.

## 6. Conclusion

In conclusion, the IL-6 level, CD4^+^ T cell percentage, NK cell percentage, and CD4^+^/CD8^+^ can be used as biological indicators to predict death in children with IAE, and the combination of these four indicators is more sensitive and has higher predictive value than one indicator alone. Our results can help clinicians deepen their understanding of the immune status of children with IAE and have reference value for evaluating the severity of children with IAE.

## Figures and Tables

**Table 1:** Comparison of the indicators between the two groups.

Characteristics and indicators	Survival group (*N* = 59)	Death group (*N* = 17)	*p*
Sex (M:F)	38:21	10:7	0.670
Age (months)	48.0 (21.0, 84.0)	49.0 (20.5, 75.0)	0.640
Underlying disease (%)	12 (20.3)	6 (35.3)	0.340
Fever days (days)	3 (1.5, 6.0)	3 (2.0, 7.0)	0.580
Influenza type			
A	46 (78.0)	11 (64.7)	0.427
B	13 (22.0)	5 (29.4)	0.759
A and B	0	1 (5.9)	—
Complication			
Sepsis (%)	6 (10.2)	4 (23.5)	0.304
Respiratory system (%)	32 (54.2)	17 (100)	0.004
Circulatory system (%)	8 (13.6)	12 (70.6)	<0.001
Digestive system (%)	14 (23.7)	13 (76.5)	<0.001
Hematological system (%)	7 (11.9)	8 (47.1)	0.004
Symptom			
Fever (%)	59 (100)	17 (100)	—
Cough (%)	35 (59.3)	8 (47.1)	0.369
Dyspnea (%)	8 (13.6)	7 (41.2)	0.030
Emesis (%)	23 (40.0)	8 (47.1)	0.551
Diarrhea (%)	3 (5.1)	2 (11.8)	0.672
Laboratory test index			
IL-6 (pg/mL)	101.5 (27.9, 211.5)	404.0 (256.3, 1228.1)	<0.001
Percentage of CD4^+^ T cells (%)	37.0 ± 8.1	27.4 ± 6.6	<0.001
Percentage of CD8^+^ T cells (%)	23.0 (20.0, 26.3)	22.0 (15.4, 26.0)	0.36
CD4^+^/CD8^+^	1.7 (1.4, 1.9)	1.2 (1.0, 1.6)	0.01
Percentage of NK cells (%)	11.0 (7.2, 13.8)	15.9 (10.7, 28.1)	0.003

Abbreviations: F, female; IL-6, interleukin-6; M, male; NK, natural killer.

**Table 2:** Comparison of IL-6, CD4^+^ T cell, NK cell, and CD4^+^/CD8^+^ ROC curves.

Predictive indicators	Cut-off	AUC	95%CI	Sensitivity	Specificity	*p* (vs. IL-6)	*p* (vs. CD4^+^ T cell)	*p* (vs. NK cell)	*p* (vs. CD4^+^/CD8^+^)
IL-6	225.3	0.812	0.706–0.892	82.4	78.0	—	—	—	—
CD4^+^ T cell	31.1%	0.810	0.704–0.891	76.5	74.6	0.984	—	—	—
NK cell	12.9%	0.740	0.627–0.834	70.6	69.5	0.464	0.379	—	—
CD4^+^/CD8^+^	1.33	0.706	0.590–0.805	58.8	78.0	0.220	0.154	0.742	—
Combined assay	0.117	0.870	0.774–0.936	94.1	67.8	0.378	0.039	0.035	0.041

Abbreviations: AUC, area under the curve; CI, confidence interval; IL-6, interleukin-6; NK, natural killer; ROC, receiver operating characteristic.

## Data Availability

The data that support the findings of this study are available on request from the corresponding author. The data are not publicly available due to privacy or ethical restrictions.
